# Rapid Development of an Ovarian Disease

**Published:** 1829-07-01

**Authors:** 


### XLIX.

### Rapid Development of an Ovarian Disease—Unsuccessful Operation.

A lady about 25 years of age, came up from the country, where she had been a short time as a teacher in an academy, in consequence of an interruption of the catamenia, and some enlargement of the lower part of the abdomen. She had been in unusual good health just before this stoppage of the menses, though she had been for years habitually costive. On examination, no tumour could be felt, though the abdomen was certainly larger than natural. There was little pain in any part of the abdomen, but a sense of distention that was very uncomfortable. The urine was scanty and lateritious. We need not detail the treatment, which was varied, but never, for an instant checked the progress of the complaint. In a few weeks the abdomen got much larger, and a tumour could be felt in one side. The pains in various parts gradually but progressively increased, and in the course of ten months from the commencement of the disease, the abdomen had got to a very great size, and the tumour was lost in what appeared to be a general effusion into the peritoneal cavity. As the unfortunate lady, who was attended by Dr. Locock, Dr. Johnson, and Mr. Bagster, could no longer bear the distention and pain, and as there was the greatest difficulty in procuring a passage through the bowels by any means, a trocar was pushed in near the umbilicus, and seven or eight pints of thin gelatinous fluid drawn off. As the abdomen subsided, the attendants were able to detect several tumours in different parts of the belly; but whether the fluid came from a large cyst, or from the general cavity, was doubtful. From the moment of the operation the sufferings of this unfortunate lady were increased tenfold, and during the remainder of her life (seven or eight weeks) exceeded any that were ever witnessed by the medical attendants. The sac, whence the fluid came, now evidently became inflamed and soon filled again with fluid of a different character. Death put a period to unheard-of sufferings, and released from protracted agonies an interesting and most patient young lady.

On dissection, one immense cyst filled with muco-purulent fluid seemed to occupy the whole abdomen. On removing twelve or fourteen pints, the internal surface of the cyst was found covered, in many places, with false membranes, the product of the inflammation which succeeded the operation. A chain of diseased ovarian cysts, in irregularly-shaped masses of various degrees of density, rose from the right ovarium, spread up under the liver, crossed over into the left side, and went down to the hollow of the ilium. They had so compressed the liver and intestines, that the former was reduced to one-fifth of its original size, and the intestines were so squeezed up against the diaphragm that it was difficult to imagine how any fæcal matters could possibly pass along them. The chain of ovarian tumours was divided into innumerable cysts containing an infinite variety of more or less fluid matters. Some of them were almost solid. The large cyst which had been tapped formed a part of this chain of cysts—and was, in fact, the master-cyst. The uterus was very small—and the left ovarium was bound down by adhesive inflammation to the contiguous parts.

The rapid progress of this dreadful and fatal disease was remarkable. Not quite twelve months before this lady's death, she was in apparent health. This activity of the disease may account for the bad effects which followed the operation ; for, in general, there is no active inflammation set up by puncturing a cyst in slowly advancing ovarian dropsy.

